# Proteomic analysis of *Chromobacterium violaceum* and its adaptability to stress

**DOI:** 10.1186/s12866-015-0606-2

**Published:** 2015-12-01

**Authors:** Diogo Castro, Isabelle Bezerra Cordeiro, Paula Taquita, Marcos Nogueira Eberlin, Jerusa Simone Garcia, Gustavo Henrique M. F. Souza, Marco Aurélio Zezzi Arruda, Edmar V. Andrade, Spartaco A. Filho, J. Lee Crainey, Luis Lopez Lozano, Paulo A. Nogueira, Patrícia P. Orlandi

**Affiliations:** Instituto Leônidas e Maria Deane – ILMD- Fiocruz, 476 Teresina St., 69057-070 Manaus, AM Brazil; Universidade Estadual de Campinas, Institute of Chemistry, Thomson Mass Spectrometry Laboratory PO and Spectrometry, Sample Preparation and Mechanization Group (GEPAM), 13084-971, Campinas, SP Brazil; Waters Corporation, 125 Alameda Tocantins, Alphaville, 06455-020 Barueri, SP Brazil; Biotechnology Laboratory/ Universidade Federal do Amazonas, 3000 Rodrigo Octávio Av., 69077-000 Manaus, AM Brazil; Universidade Estadual do Amazonas, 3578 Djalma Batista Av., 69050-010 Manaus, AM Brazil

**Keywords:** *Chromobacterium violaceum*, MALDI-tof, Stress-conditions, Biosynthetic pathways, Transporters, Receptors

## Abstract

**Background:**

*Chromobacterium violaceum (C. violaceum)* occurs abundantly in a variety of ecosystems, including ecosystems that place the bacterium under stress. This study assessed the adaptability of *C. violaceum* by submitting it to nutritional and pH stresses and then analyzing protein expression using bi-dimensional electrophoresis (2-DE) and Maldi mass spectrometry.

**Results:**

*Chromobacterium violaceum* grew best in pH neutral, nutrient-rich medium (reference conditions); however, the total protein mass recovered from stressed bacteria cultures was always higher than the total protein mass recovered from our reference culture. The diversity of proteins expressed (repressed by the number of identifiable 2-DE spots) was seen to be highest in the reference cultures, suggesting that stress reduces the overall range of proteins expressed by *C. violaceum*. Database comparisons allowed 43 of the 55 spots subjected to Maldi mass spectrometry to be characterized as containing a single identifiable protein. Stress-related expression changes were noted for C*. violaceum* proteins related to the previously characterized bacterial proteins: DnaK, GroEL-2, Rhs, EF-Tu, EF-P; MCP, homogentisate 1,2-dioxygenase, Arginine deiminase and the ATP synthase β-subunit protein as well as for the ribosomal protein subunits L1, L3, L5 and L6. The ability of C*. violaceum* to adapt its cellular mechanics to sub-optimal growth and protein production conditions was well illustrated by its regulation of ribosomal protein subunits. With the exception of the ribosomal subunit L3, which plays a role in protein folding and maybe therefore be more useful in stressful conditions, all the other ribosomal subunit proteins were seen to have reduced expression in stressed cultures. Curiously, *C. violeaceum* cultures were also observed to lose their violet color under stress, which suggests that the violacein pigment biosynthetic pathway is affected by stress.

**Conclusions:**

Analysis of the proteomic signatures of stressed *C. violaceum* indicates that nutrient-starvation and pH stress can cause changes in the expression of the *C. violaceum* receptors, transporters, and proteins involved with biosynthetic pathways, molecule recycling, energy production. Our findings complement the recent publication of the *C. violeaceum* genome sequence and could help with the future commercial exploitation of *C. violeaceum.*

**Electronic supplementary material:**

The online version of this article (doi:10.1186/s12866-015-0606-2) contains supplementary material, which is available to authorized users.

## Background

*Chromobacterium violaceum* is a soil and water borne Gram-negative β-proteobacterium that is found in tropical and subtropical regions and produces the intense purple pigment violacein [1, 2]. Although human infections with *C. violaceum* have been reported worldwide (reviewed in [3, 4]) opportunistic infections caused by the species are rare and research interest in the species has historically focused on its potential biotechnological and pharmaceutical applications [5–9]. Prior to the publication of the complete *C. violaceum* genome, most research was centered on its colored pigment violacein, which is an amino acid chain with antimicrobial and dermatological properties that the bacteria secretes. However, since the publication of the genome of *C. violaceum*, which revealed the existence of a diverse range of genes that could be involved in a variety of biotechnologically-relevant metabolic pathways, research interest in the species has broadened.

Analysis of the *C. violaceum* genome has identified genes involved in alternative pathways for mercury-free gold-solubilization and energy production and genes that have the potential to be involved with the decomposition of plastics as well as genes associated with halogenated compound production pathways, the existence of which suggests that *C . violaceum* could play a role in bioremediation of contaminated soils [6, 7]. Similarly, genomic analysis has also revealed the existence of genes likely to be involved with the bacteria’s response to heat-stress response and an extensive repertoire of genes likely to be related to the ability of *C. violaceum* to adapt to a wide range of environmental conditions, such as UV radiation [1, 10, 11].

Although stress-related gene pathways are less directly connected with biotechinologically exploitable functions than some of the other recently identified genes, understanding how these stress-related genes function and are regulated could nevertheless prove useful for the future manipulation and commercial exploitation *C. violaceum.*

*Chromobacterium violaceum* can be found naturally occurring in a diverse range of environmental conditions. Some strains of *C. violaceum* grow naturally in tropical areas, whereas others have been isolated in Antarctica [12, 13]. In the Brazilian Amazon region, *C. violaceum* is a major component of the tropical soil microbiota, and is also found abundantly in the Rio Negro river [2, 5, 6, 14, 15]. Laboratory studies have illustrated the remarkable adaptability of C*. violaceum* to changes in environmental conditions, like iron influence and stressful growth temperatures, to determine the specific enzymes and elements involved in the genetic regulation [15, 16].

Building on the recent discoveries from the genome *C. violaceum* project, this study examined the protein expression patterns of *C. violaceum* when it is grown under pH stress and nutrient-starvation conditions. Raw protein extracts obtained from the various cultures have been ran on SDS-PAGE gels to produce stress-related protein expression profiles. The Intensities of protein spots observed in these 2-DE gels were assessed via comparison with a reference gel and certain spots were selected for analysis. Mass spectrometry was performed to identify whether differential protein profiles might reveal information concerning the strategies that *C. violaceum* uses to adapt to stress.

## Methods

### Bacteria growth and protein extraction

*Chromobacterium violaceum* strain ATCC12472 was reactivated in LB broth under gentle agitation, and streaked onto LB-agar plates to ensure its purity. A single colony was grown at 35 °C using the same LB broth as the original source. In the pH stress assays, a small aliquot was transferred in 100 ml of LB broth adjusted to low (4.0); middle (7.0) or high (9.0) pH. In the nutrient-starvation stress assays, a small aliquot was transferred on minimal salts microbial growth medium (M9 medium, Sigma-Aldrich, Brazil). After 7 h under gentle agitation, the bacterial cultures were harvested by centrifugation at 12,000 g for 15 min at 4 °C and washed in 75 mM Tris–HCl pH 7.0 buffer. A hundred milligrams of bacterial pellet was washed twice in 1 mL of Milli-Q water with 2 mM PMSF (Phenylmethylsulfonyl Fluoride, Thermo Scientific) and a Protease Inhibitor Cocktail (PIC - Amersham Bioscience) following manufacturer recommendations. The aliquots were stored at −80 °C prior to use.

The stored bacterial pellet was lysed in IPG buffer (Amershan Bioscience) composed of PIC, 8 M urea, 2 % [(3-cholamidopropyl)-dimethylammonio]-1-propanesulfonate (CHAPS), 100 mM ditiotreitol (DTT) and 80 mM citric acid. After centrifugation at 12,000 g for 20 min at 25 °C, the lysates were precipitated with 500 μL of acetone for 1 h at room temperature, and centrifuged at 12,000 g for 10 min at 25 °C to remove the cell debris. The precipitates were washed three times in acetone (80 %) and dried at room temperature. Total protein was quantified using a commercial colorimetric kit based on the Lowry method following the manufacturer’s recommendations (BioAgency), and stored at −20 °C until the isoelectric focusing experiments were performed.

### 2-DE gel and spot quantification

The first-dimension separation began with isoelectric focusing (IEF) using the IPGPhor3, an integrated IEF-system (GE Healthcare). In brief, 250 μg of protein were rehydrated in DeStreak Rehydration Solution (Amersham Bioscience) in 0.5 % IPG buffer (GE Healthcare). Samples were applied to a pH 3–11 NL, immobilized pH gradient (IPG) by passive rehydration using the IPGPhor3 for 10 h at 20 °C. The focusing conditions were: 150 V (2 h), 300 V (2 h), 1000 V (gradient for 4 h), 8000 V (gradient for 2 h), and 8000 V (2 h). In second-dimension separation, SDS-PAGE electrophoresis, IPG strips were soaked with equilibration buffer 1 (75 mM Tris–HCl buffer pH 8.8, 6 M urea, 2%SDS, 29.3 % glycerol, 1 % DTT) for 8 min and removed, and then soaked in equilibration buffer 2 (6 M urea, 2 % SDS, 29.3 % glycerol, 2.5 % iodoacetamide, 75 mM Tris–HCl pH 8.8) for 12 min. Lastly, the IPG strips were soaked in 75 mM Tris–HCl pH 6.8 for 2 min and placed on 12.5 % (*w/v*) polyacrylamide resolving gels in a SE600Rub System (GE Healthcare) under 10 mA/gel for 45 min. The polyacrylamide gels were fixed using 10 % acid acetic and 40 % methanol, followed by staining with Colloidal Coomassie Blue (8 % ammonium sulfate, 0.8 % phosphoric acid, 0.08 % Coomassie Blue G-250 and 20 % methanol) [16]. Gel images were captured by scanning (Image Scanner, GE Healthcare), and analyzed by Image Master Platinum software (Version 6). Three reproducible gels (over 70 % in similarity) were produced to correspond with at least two independent extraction procedures from each experimental condition [16]. The spot detection parameters were determined by the ImageMaster Platinum software to detect spots in non-DIGE gels. In brief, ImageMaster Platinum software used a smooth-by-diffusion algorithm to detect the *minimal area* defining a spot. The software used the *saliency* parameter, expressed in number of pixels, to estimate the *threshold* that distinguishes a real spot from the background of the gel. Areas were then split into as many overlapping spots as possible. The spots were quantified automatically based on two final parameters, *intensity* and *area*. The spot *intensity* was relative to background; a minimum pixel value above the spot neighborhood was defined as a unit of intensity. Finally, the ImageMaster quantified the *intensity* (*I*) of a spot based on the pixel units per central area, corresponding to 75 % of an encircled spot. To assess the influence of pH and nutrient-starvation stresses on protein expression, the Intensities of 15 spots observed in all 2-DE gels were assessed via comparison with a reference gel and certain spots were selected for analysis.

### Preparation of spots to mass spectrometry

For the mass spectometry analysis, several spots were selected based on diffrrences in intensity between spots grown under pH 4.0, pH 9.0, or M9 medium, and spots from the reference gel (grown under pH 7.0.). The selected spots were excised from the polyacrylamide gels, and disrupted in order to digest proteins prior to mass spectrometry. In brief, the extracts were digested with Trypsin Porcine Pancreas (Sigma) using the Montage In-Gel DigestZP Kit (*ZipPlate,* Millipore, USA) following the manufacturer’s recommendations.

### Mass spectrometry

Trypsin-digested samples were applied to the microplate using the dried droplet method. A matrix solution was prepared using α-cyano-4-hydroxycinnamic acid in a 1:1 (*v/v*) acetonitrile/H_2_O solution, containing 0.1 % (*v/v*) TFA (Trifluoroacetic acid). The matrix was added to samples with a total volume of 1.2 μL and allowed to dry at room temperature [17].

The mass spectra were acquired in a MALDI Q-TOF Premier mass spectrometer (Waters, Micromass, Manchester, UK). The mass spectra were obtained with a solid- state laser in a positive mode (LDI+) using the following parameters: laser firing rate and repetition rate of up to 200 Hz, 100 shots per spectra; laser wavelength 355ηm, pulse width 3ηs, pulse energy 100 μJ, peak power 34 kW; beam divergence and full angle <2 mrad. Real-time calibration was performed with Lock Mass correction using a mixture of PEG (poly[ethylene glycol]) oligomers (PEG 600, 1000, 1500 and 2000). The main parameters were: mass range from 880.0 to 4000.0 Da, peak detection threshold for MS/MS of 150.0, mass threshold of 80.0 Da, inter-scan time of 0.1 s, resolution of 10,000 in “V” mode, trigger threshold of 700 mV, signal sensitivity of 80 mV, and microchannel plate photomultiplier (MCP) set to 2200 V. Each spectrum was collected in a 1 s scan, and the spectra were accumulated for 2 min. The instrument was controlled by Mass Lynx v.4.1 software. Protein identification was achieved via database search using the peptide peak (masses and intensities) for mass spectra post-processing through Protein Lynx Global Server v.2.3 (Waters Corporation, UK). The mono-isotopic peak lists were processed using the following parameters: one missed cleavage, tryptic digestion, carbamide methylation and cysteine modification with search error tolerance set at 5 ppm with a [M + hrs]^+^ charge state [18]. The protein data was compiled by Swiss Prot using information about the *C. violaceum* genome from the Expasy Databank. The protein data was submitted to the Protein Lynx Global Server program [19–21].

## Results and discussion

### *Chromobacterium violaceum* growth patterns under stress

**Fig. 1:**
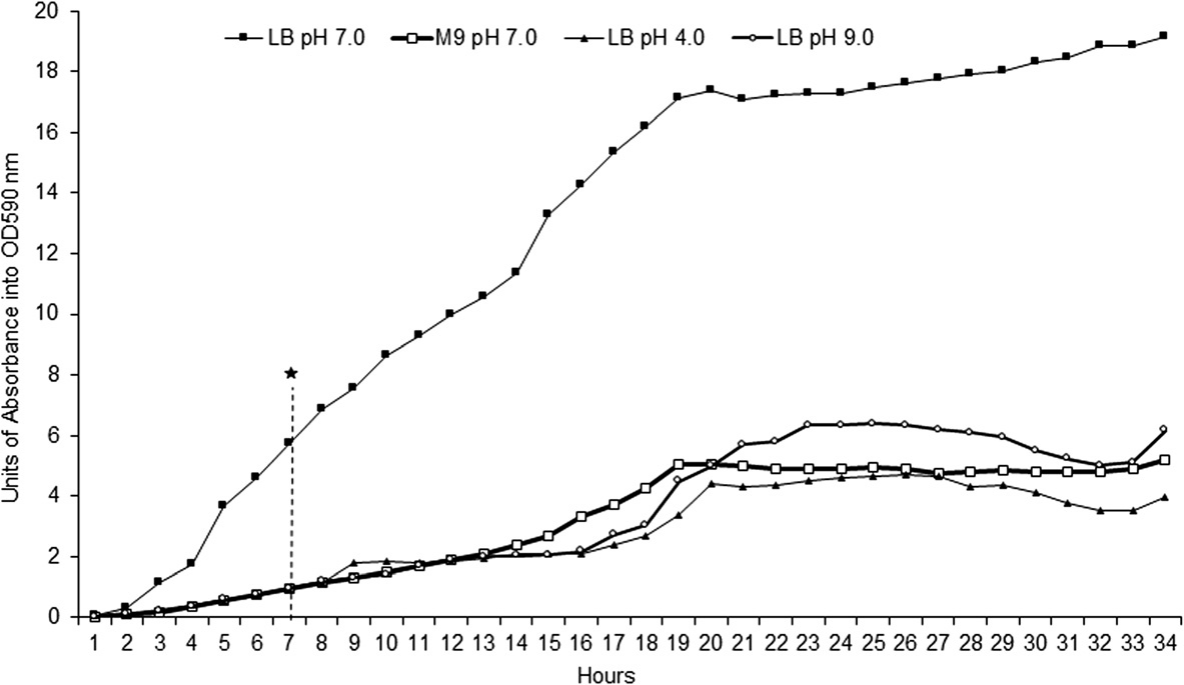
Growth curve of *Chromobacterium violaceum*: Long cultures of *C. violaceum* grown in rich medium (LB) and nutrient-poor medium (M9) for 34 h and rotated at 35 °C to determine the extent of the exponential and stationary phases. *Asterisk*: The time chosen as representative of the exponential growth phase in all cases

*Chromobacterium violaceum* (ATCC12472) was grown under gentle agitation at 35 °C for 34 h to establish the exponential and stationary phases of a reference culture and a set of three differently stressed cultures (Fig. 1). The growth curves of *C. violaceum* displayed the same behavior in all conditions: growth entered the exponential phase after 4 h, and this phase persisted until 19 h, at which point the stationary phase started. As expected, *C. violaceum* grew best in the pH neutral nutrient-rich (LB) media. Under these reference conditions the bacteria culture exhibited a dark-violet metallic sheen, the optical density of which was observed to be 17 units at 590 nm. Under nutrient-starvation and pH stress, *C. violaceum* lost its purplish pigmentation. Despite the remarkable adaptability of *C. violaceum* to environmental changes, pH stress proved detrimental to its growth. Under pH stress, *C. violaceum* exhibited low growth even in rich medium. The growth patterns of *C. violaceum* observed in this study are in agreement with the growth curves of other species submitted to harsh environmental conditions [22, 23].

### *Chromobacterium violaceum s*tress-related protein expression profiles

**Fig. 2:**
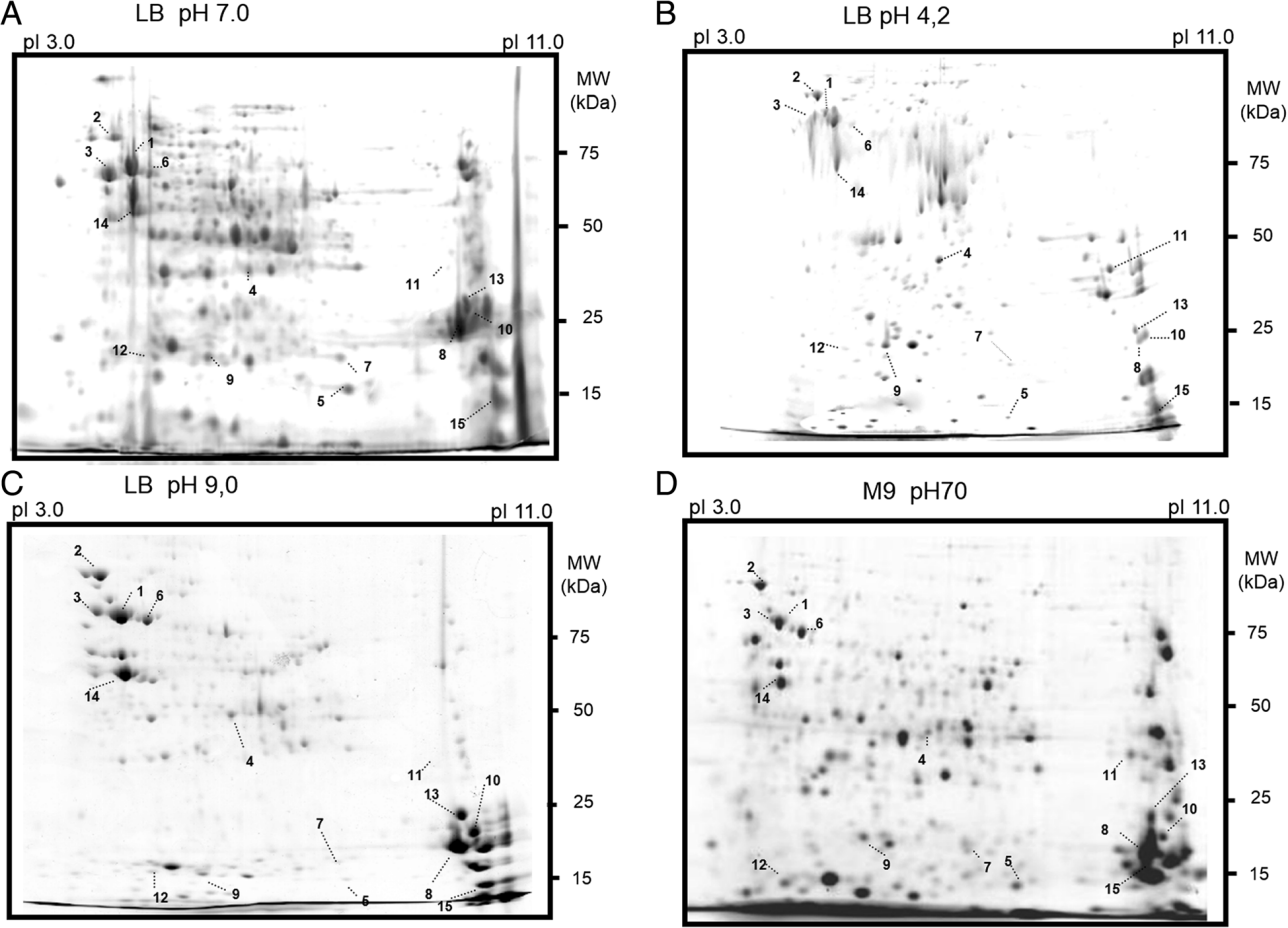
2-D gel profiles of *C. violaceum* expressed proteins under stresses. *C. violaceum* was cultured for 7 h of exponential growth under stress-conditions: **a** Luria Bertani (LB) medium at pH 7.0 (defined as reference gel); **b** LB at pH 4.0; **c** LB at pH 9.0 and **d** M9 minimum medium at pH 7.0. The expressed proteins were submitted to isoelectric focusing on pH 3.0–11.0 gradient strips and split onto 12 % polyacrylamide gels. Selected spots for each condition (numbered 1 to 20) were identified by MALDI/MS

Table 1 shows the total protein extract obtained from 100 mg of bacterial cells and shows that overall protein content of *C. violaceum* is higher in stressed cultures than it is in unstressed cultures. Spot counts were compared in 2-DE to assess the effect of pH and nutrient-starvation stress on protein expression. Although the total protein production was highest in stressed cultures, *C. violaceum* growth in reference conditions, showed by far the greatest diversity in the types of protein expressed (see Fig. 2a). More than 300 spots were identifiable from *C. violaceum* cultures grown under these reference conditions (Fig. 2a). *Chromobacterium violaceum* growth in pH 4.0 produced profiles with 238 identifiable spots, and growth in pH 9.0 resulted in 244 such spots. *Chromobacterium violaceum* 2-DE protein profiles for bacteria grown under nutrient-starvation also contained 238 identifiable spots. In some cases, distinct stress-specific spot profiles were clearly identifiable and maintained over time (see Additional file 1: Figure S1). Similar data have been observed with *Brucella suis (B. suis)* when it was submitted to long-term nutrient-starvation. For *B. suis* less than half the 2-DE spots that could be identified in reference culture profiles could be seen in stressed culture profiles-- suggesting that under stress, *B. suis* reduces the diversity of the proteins it expresses even more markedly than *C. violaceum* [22]. Interestingly, nutritional stress profiles were different from pH stress profiles, suggesting that the *C. violaceum* protein expression response to stress is tailored to specific environmental stresses [6, 7, 10].

**Table 1:** Total protein mass obtained from a 100 mg of bacterial pellet after 7 h of growth

Culture conditions	Total protein mass (μg)	Number of spots identifiable in 2-DE
LB pH 7.0	24.1 ± 0.3	>300
LB pH 4.0	24.0 ± 0.2	238
LB pH 9.0	23.0 ± 0.3	244
M9 pH 7.0	35.8 ± 0.3	238

To better understand which proteins were influenced by pH and nutrient-starvation stresses, spots in 2-DE with different pixel units were selected for mass spectrometry. Fifty-five spots were submitted for comparison against the mass spectrometry databank, and 43 of them were identified (Table 2). Despite the advanced state of proteomic and genomic technologies, we were unable to classify 12 proteins [19, 20, 23]. Interesting most of the spots that were selected contained just a single protein (Fig. 2, Table 2).

**Table 2:** Protein identification of *C. violaceum* by MALDI/MS

Description	Theorical		Experimental		Score
	mW (Da)	pI	mW (Da)	pI	
*C.violaceum* 50S ribosomal L1 protein^a^	22,226	10.3301	22,240.16	9.90	8.2941
*C.violaceum* 50S ribosomal L3 protein^a^	23,946	10.1124	23,961.41	9.66	8.2941
*C.violaceum* 50S ribosomal L4 protein^a^	22,874	10.3385	22,888.02	9.93	8.2941
*C.violaceum* 50S ribosomal L5 protein^a^	20,292	9.7899	20,305.55	9.44	8.2941
*C.violaceum* 50S ribosomal L6 protein^a^	18,784	10.1521	18,795.70	9.68	8.2941
*C.violaceum* acetyl-CoA acetyltransferase ^a^	40,094	6.3565	40,118.74	6.33	8.2941
*C.violaceum* acuC-aphA family histone deacetylase	32,920	5.7805	32,940.88	5.73	8.2941
*C.violaceum* alpha chain nitrate reductase	137,284	6.2869	137,371.63	6.28	8.2941
*C.violaceum* amino acid permease	46,898	7.7271	46,928.90	8.21	8.2941
*C.violaceum* arcA arginine deiminase	46,177	5.9075	46,206.68	5.93	8.2941
*C.violaceum* beta subunit ATP synthase	50,024	4.9332	50,055.78	5.10	8.2941
*C.violaceum* chromate ion transporter protein	45,072	11.0618	45,100.87	10.58	8.2941
*C.violaceum* depB protein	162,060	4.7681	162,162	4.81	8.2941
*C.violaceum* dismutase ^a^	21,576	5.8577	21,589.99	5.86	8.2941
*C.violaceum* dnaK chaperone protein ^a^	69,079	4.7521	69,121.62	4.94	8.2941
*C.violaceum* elongation factor P protein	20,893	4.5956	20,906.61	4.79	8.2941
*C.violaceum* factor Tu	43,045	4.9378	43,071.71	5.11	8.2941
*C.violaceum* flagellar hook protein E	43,040	4.3434	43,066.86	4.54	8.2941
*C.violaceum* fructose bifosfato aldolase ^a^	38,294	5.5995	38,318.41	5.56	8.2941
*C.violaceum* Glutaminyl-tRNA synthetase	64,332	5.395	64,371.73	5.45	8.2941
*C.violaceum* groel 2 chaperonin ^a^	57,382	4.8968	57,417.73	5.09	8.2941
*C.violaceum* haloacid dehalogenase	26,514	5.798	26,530.92	5.85	8.2941
*C.violaceum* homogentisate 1 2 dioxygenase	42,386	5.8835	42,413.36	5.89	8.2941
*C.violaceum* large subunit Glutamate synthase	161,839	5.9496	161,940.51	6.02	8.2941
*C.violaceum* membrane protein	24,587	9.7804	24,604.19	9.69	8.2941
*C.violaceum* methyl accepting transducer chemotaxis transmembrane protein	59,066	5.2366	59,102.16	5.37	8.2941
*C.violaceum* multidrug efflux protein ^a^	49,197	9.8397	49,229.02	9.72	8.2941
*C.violaceum* multidrug resistance transmembrane protein	54,368	10.8052	54,402.42	10.57	8.2941
*C.violaceum* ornithine carbamoyl transferase	37,739	5.8079	37,763.68	5.96	8.2935
*C.violaceum* oxaA Inner membrane protein	60,494	9.3199	60,532.79	9.09	8.2935
*C.violaceum* phasin protein ^a^	19,460	7.7754	19,471.52	6.84	8.2941
*C.violaceum* phbF protein	21,440	4.7708	21,454.60	4.97	8.2941
*C.violaceum* phosphoenolpyruvate phosphotransferase ^a^	89,323	5.2925	89,378.22	5.43	8.2941
*C.violaceum* pily1 related type 4 fimbrial biogenesis protein	111,408	4.9526	111,476.45	5.03	8.2941
*C.violaceum* putative protein	59,467	9.8919	59,503.50	9.74	8.2941
*C.violaceum* resistance protein	42,838	9.2531	42,866.11	9.19	8.2941
*C.violaceum* rhs related protein	123,858	5.2976	123,934.25	5.42	8.2941
*C.violaceum* secretion protein	45,487	10.2636	45,482.62	9.97	8.2941
*C.violaceum* sodium dependent transporter protein	51,277	9.9326	51,310.86	9.63	8.2941
*C.violaceum* tail fiber related protein	67,404	5.0552	67,445.36	5.23	8.2839
*C.violaceum* TonB dependent receptor	85,159	6.6671	85,211.14	6.61	7.6150
*C.violaceum* transmembrane drug efflux pump protein	111,151	9.4457	111,220.34	9.27	8.2914
*C.violaceum* triacylglycerol lipase	32,150	7.9748	32,170.45	7.73	8.2931
*C.violaceum* uncharacterized protein	19,630	5.4373	19,641.70	5.61	8.2941
*C.violaceum* uncharacterized protein	27,304	5.9343	27,321.45	5.71	8.2941
*C.violaceum* uncharacterized protein	59,467	9.8919	59,503.50	9.74	8.2941
*C.violaceum* uncharacterized protein	20,756	6.1716	20,769.32	6.15	8.2930
*C.violaceum* uncharacterized protein	21,227	6.7355	21,240.96	6.37	8.2941
*C.violaceum* uncharacterized protein	23,771	6.343	23,786.15	6.12	8.2941
*C.violaceum* uncharacterized protein	37,473	8.2407	37,465.53	8.43	8.2941
*C.violaceum* uncharacterized protein	60,019	4.6575	60,054.62	4.84	8.2941
*C.violaceum* uncharacterized protein	92,113	5.298	92,138.40	5.42	8.2941
*C.violaceum* uncharacterized protein	19,959	8.9654	19,972.03	7.98	8.2941
*C.violaceum* uncharacterized protein	60,732	9.6696	60,769.16	9.50	8.1529
*C.violaceum* uncharacterized protein	101,364	10.8481	101,425.36	10.38	8.2941

Note: *MW* molecular weight and Daltons (Da) the mass unity, *pI* isoelectric point. ^a^same proteins shared in Cordeiro and collaborators [13]

**Fig. 3:**
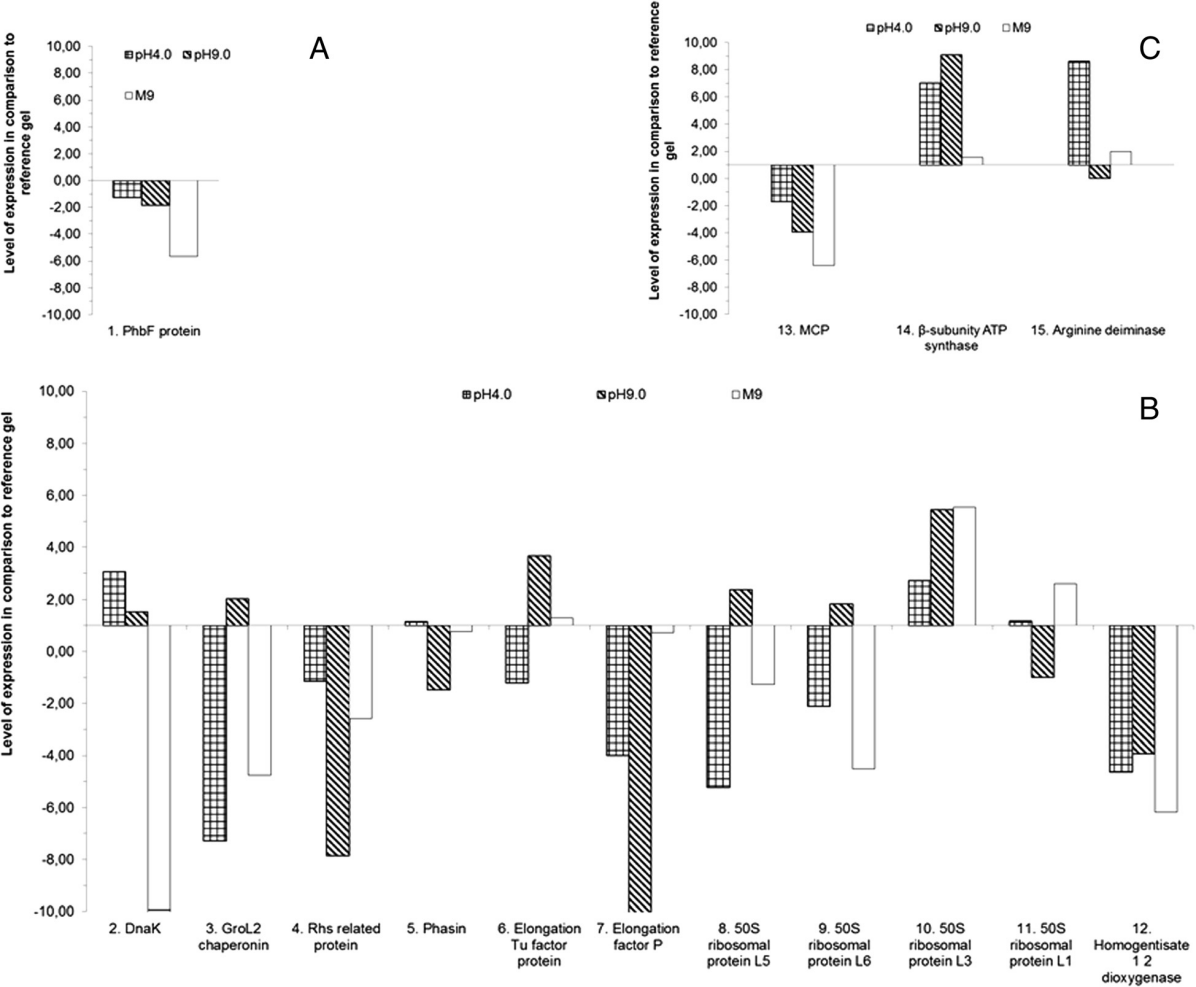
Identification of expressed proteins by MALDI/MS. *C. violaceum* proteins under pH stress: **a** Proteins associated with bacterial responses; **b** Biosynthesis related proteins; **c** Energy and metabolism related proteins

Some of the identifiable proteins seem to be correlated with nutrient-starvation survival strategies including biosynthesis, molecules recycling and energy production [22]. The identified spots included proteins belonging to energetic metabolism, elements of biosynthetic pathways like chaperones, ribosomal proteins, transporters, and receptors (see Table 2). Fifteen spots corresponding to 15 characterized proteins were classified into three major functional groups that were analyzed quantitatively (See Fig. 3a–c). The first functional group, referred to here as the “molecule recycling group”, was represented with just one protein: the polyhydroxy butyrate protein (PhbF) (Fig. 3a). The second group, referred to here as the “biosynthesis protein group” was comprised of proteins, such as elongation factors and ribosomal subunits (Fig. 3b). The third group protein group was comprised of proteins related to energy production and metabolism and is referred to here as the “energy related” protein group (Fig. 3c).

**Fig. 4:**
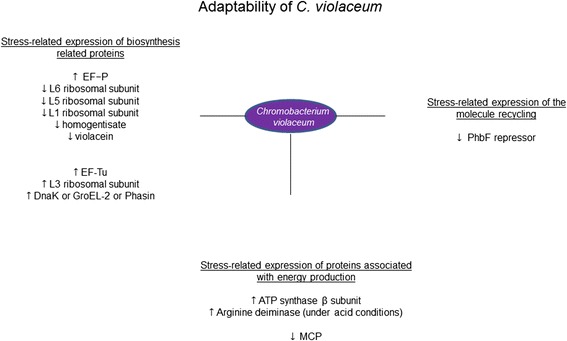
Spectrum of stress-specific proteins of *Chromobacterium violaceum*. Proposition of coordinated responses based on expressed proteins by *C. violaceum*

### *S*tress-related expression of the molecule recycling protein PhbF

The *C. violaceum* PhbF protein is probably involved in molecule recycling and, in this study, appeared to be produced in lower quantities under stress than when it was grown in reference conditions. The PhbF is a DNA binding protein that belongs to a family of polyhydroxy alkanoate synthesis repressors that produce polyhydroxy butyrate for intracellular granular storage [24]. In the exponential phase, *C. violaceum* reduced expression of this repressor probably to allow the production of PHB under low levels of nitrogen, phosphates or oxygen possibly because of its ability to store nutrients even in adverse environments [24–26].

### Stress-related expression of biosynthesis related proteins

Some of the proteins with stress-associated expression patterns seem to be correlated with biosynthesis of amino acids and nucleic acids. The chaperones DnaK (heat shock protein), GroEL-2 and Phasin are both highly conserved proteins involved in biosynthesis and were both more abundant in *C. violaceum* cultures grown under stress conditions. DnaK, phasin, chaperonin GroEL-2 function as accessory proteins in chaperone machines [6, 16, 27–29] and increased chaperone expression was associated with stress tolerance and response to a high concentration of iron or carbon/energy or nitrogen nutrient-starvation [15, 30]. Our data is in agreement with the role of these proteins in protection against damage caused by pH and nutrient-starvation stresses [31–34]. The Rhs-related proteins (Rearrangement hotspot – related protein) are widely distributed in bacteria and eukaryotes and it has been proposed that expression of the Rhs components increases ribosomal gene expression [35–37]. In the exponential phase, *C. violaceum* showed reduced expression of Rhs-related proteins which would repress protein biosynthesis even in adverse environments. The synthesis of EF-Tu (elongation factor thermo unstable) and EF-P (Bacterial elongation factor P) appeared to be similar in the reference and nutrient-starved cultures, but appeared to be reduced under pH stress. EF-Tu is important for translational accuracy in protein synthesis [38–40]. In a study on the stress resistance of *Vibrio cholerae*, the stabilization of ET-Tu with heat-shock chaperone demonstrated that it is a highly sensitive protein that significantly enhances the stress resistance of this bacterium [40]. It appears that EF-Tu acts as a down-regulator of translational control for most protein synthesis during nutrient-starvation while allowing the synthesis of nutrient-starvation-induced proteins [41, 42]. The down regulation of EF-P in nutrient-starvation and pH stress conditions suggests that *C. violaceum*’s protein expression maybe controlled by the same universal bacterial factor [39, 40, 42, 43]. Consistent with previous findings, a suite of ribosomal proteins were also observed to show stress-related expression patterns in *C. violaceum*. In this study, the synthesis of L6, L5, L3 and L1 ribosomal subunits we all observed to vary with stress conditions, supporting the notion that the bacteria’s core cellular mechanics are well-adapted to changes in nutrient and pH conditions. *Chromobacterium violaceum’s* increased synthesis of ribosomal L3 subunit, which has a role in minimizing protein miss-folding, is consistent with what has been demonstrated for other Gram-negative bacteria [44, 45]. Belonging to the large prokaryotic ribosomal subunit, the observed elevated nutrient-starvation-induced ribosomal L3 subunit production may be the bacteria’s response to sub-optimal protein folding conditions [6, 46]. Relative to the cultures grown under reference conditions, the synthesis of the other ribosomal subunits appears to be lower (see Fig. 3b), suggesting that *C. violaceum* reduces overall protein production as a response to stress. It is important to note, however, that the ribosomal protein spots are far more intense feature of stress profiles after 18 h of growth than they are after 34 h (see Additional file 1: Figure S1), suggesting their down-regulation is delayed and not an immediate shock-response to stress. This may help to explain why overall protein content was higher in nutrient starvation stress than in the reference culture (see Table 1) and may be a sign that the bacteria reduce overall protein production as an adaptation to stressful growth conditions, but that overall protein production increases initially as the bacteria transitions to its new harsher growth environment.

The color change noted for all stressed cultures of *C. violaceum* is almost certainly explained by the stressed-cultures’ loss of the violacein pigment. As the violacein of *C. violaceuim* is produced by the fusion of two tryptophan residues formed by decarboxylation, it can be seen as representing another biosynthethic pathway which is affected by stress and thus as a convenient indicator of biosynthetic activity [47]. Interestingly our study also noted a reduction of homogentisate 1,2-dioxygenase in all three stressed cultures, which maybe correlated with the bacteria’s stress-induced color change. Consistent with this notion, in other bacteria homogentisate 1,2-dioxygenase is known to catalyze the conversion of homogentisate to 4-maleylacetoacetate in the catabolism of aromatic rings [48].

### Stress-related expression of proteins associated with energy production

The methyl-accepting chemotaxis protein (MCP) is a transmembrane sensor protein associated with the bacterial mechanisms which recruit cytoplasmic components of the signaling pathway for sensing and responding to chemical changes [49]. Meier and Scharf demonstrated that a MCP-S receptor protein of the free-living bacterium *Sinorhizobium meliloti* is usually weakly expressed under normal growth conditions and speculated that it might be up-regulated when the bacteria is in its natural symbiotic chemical environment [50]. Under stress conditions *C. violaceum* has reduced expression of MCP during the exponential growth phase (see Fig. 3a), which may be to avoid the synthesis of unnecessary proteins. Arginine deiminase (Fig. 3c) participates in arginine and proline metabolism related to adaptation to low pH environments. The arginine deiminase system was found to protect bacterial cells against the damaging effects of low pH [21]. Other examples of the arginine deiminase system protecting against low pH environments are seen in *Listeria monocytogenes*, which can survive in low-pH foods and pass through the gastric barrier of its host, and in the protection of oral bacteria from acid [51, 52]. In this study, the up-regulation of arginine deiminase should be considered an indication that *C. violaceum* can adapt to low pH conditions such as those found in the Negro River, which range from pH 3.8 to 4.9 [12, 53]. Synthesis of the ATP synthase β subunit in *C. violaceum* grown under stress was also seen to be elevated in this study (See Fig. 3c). In *Escherichia coli* and bovine mitochondria [48] this protein contributes to catalytic sites in regulation of ATP synthase activity. Similarly, ATP synthase β subunit was seen to have elevated expression under glucose privation in the human hepatocellular carcinoma cell line HepG2 [54]. From analysis of the *B. suis* proteome, an increase in β-ATP synthase expression was seen to be associated with nutrient-starvation conditions [22]. The results presented here thus suggest that ATP synthase β subunit of *C. violaceum* may have the same regulatory role for adapting to ATP limitation that it has been observed to have in other prokaryotic and eukaryotic cells alike [22, 48, 54].

## Conclusions

Our data showed that under nutrient-starvation and pH stresses *C. violaceum*’s 2DE signatures are changed markedly. Under such stresses, *C. violaceum* was seen to increase its overall protein production, but appeared to reduce the diversity of proteins it synthesizes (Fig. 4). Our analysis of protein expression indicated that stress cues affect *C. violaceum* receptors, transporters, and proteins that effect energy consumption, biosynthesis and molecular recycling. Most ribosomal subunit protein production was reduced in all three stressed cultures of *C. violaceum* suggesting that the bacteria’s protein production and general biosynthesis is decreased as an adaptation to stressful growth conditions. Stressed cultures, however, also showed a notable increase in the production of the L3 ribosomal protein subunit, which probably helps the bacteria with sub-optimal protein folding conditions and could be taken as evidence that the metabolic machinery of the bacteria is capable of adapting to more hostile environmental conditions. In stressed cultures *C. violaceum* was noted to lose its distinctive color and to have reduced levels of homogentisate 1,2-dioxygenase, which is sometimes involved in catabolic biosynthetic colored pigment production. Our findings complement the recent publication of the *C. violaceum* genome sequence by enriching the available protein expression data and provide a valuable preliminary insight into the environmental adaptability of *C. violaceum*, which could help with the future commercial exploitation of C*. violaceum.*

## Additional file

Additional file 1: Figure S1. Comparison of 2-D gel profiles of *C. violaceum* expressed proteins between reference conditions and nutrient-poor medium. *Chromobacterium violaceum* expressed proteins after growth for 19 and 35 h: A) Nineteen hours of growth in LB medium at pH 7.0; B) Nineteen hours of growth in M9 poor medium; C) Thirty-five hours of growth in LB medium at pH 7.0 and D) Thirty-five hours of growth in M9 minimum medium at pH 7.0. The expressed proteins were submitted to isoelectric focusing on pH 3.0–11.0 gradient strips and split onto 12 % polyacrylamide gels. Selected spots for each condition (numbered 1 to 20) were identified by MALDI/MS. (TIFF 1890 kb)
