# Supplementary figures and images for: Proteomic analysis of Chromobacterium violaceum and its adaptability to stress
Source: BMC Microbiol. 2015 Dec 1;15:272. doi: 10.1186/s12866-015-0606-2 (PMC4666173; doi:10.1186/s12866-015-0606-2)

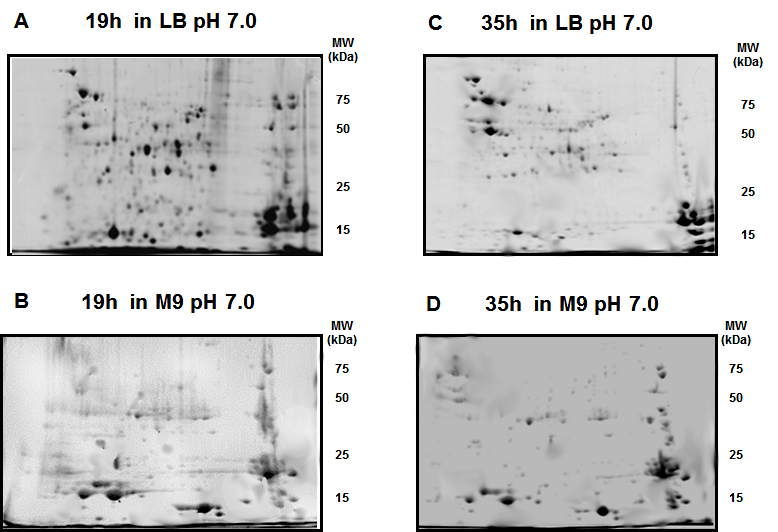

Supplement: Additional file 1: Figure S1. — Comparison of 2-D gel profiles of C. violaceum expressed proteins between reference conditions and nutrient-poor medium. Chromobacterium violaceum expressed proteins after growth for 19 and 35 h: A) Nineteen hours of growth in LB medium at pH 7.0; B) Nineteen hours of growth in M9 poor medium; C) Thirty-five hours of growth in LB medium at pH 7.0 and D) Thirty-five hours of growth in M9 minimum medium at pH 7.0. The expressed proteins were submitted to isoelectric focusing on pH 3.0–11.0 gradient strips and split onto 12 % polyacrylamide gels. Selected spots for each condition (numbered 1 to 20) were identified by MALDI/MS. (TIFF 1890 kb) [file 12866_2015_606_MOESM1_ESM.tiff]
